# The biofilm matrix destabilizers, EDTA and DNaseI, enhance the susceptibility of nontypeable *Hemophilus influenzae* biofilms to treatment with ampicillin and ciprofloxacin

**DOI:** 10.1002/mbo3.187

**Published:** 2014-07-06

**Authors:** Rosalia Cavaliere, Jessica L Ball, Lynne Turnbull, Cynthia B Whitchurch

**Affiliations:** 1 The ithree Institute, University of Technology SydneySydney, New South Wales, Australia; 2 Department of Microbiology, Monash UniversityClayton, Victoria, Australia

**Keywords:** Antibiotic, biofilm, cations, eDNA, matrix

## Abstract

Nontypeable *Hemophilus influenzae* (NTHi) is a Gram-negative bacterial pathogen that causes chronic biofilm infections of the ears and airways. The biofilm matrix provides structural integrity to the biofilm and protects biofilm cells from antibiotic exposure by reducing penetration of antimicrobial compounds into the biofilm. Extracellular DNA (eDNA) has been found to be a major matrix component of biofilms formed by many species of Gram-positive and Gram-negative bacteria, including NTHi. Interestingly, the cation chelator ethylenediaminetetra-acetic acid (EDTA) has been shown to reduce the matrix strength of biofilms of several bacterial species as well as to have bactericidal activity against various pathogens. EDTA exerts its antimicrobial activity by chelating divalent cations necessary for growth and membrane stability and by destabilizing the matrix thus enhancing the detachment of bacterial cells from the biofilm. In this study, we have explored the role of divalent cations in NTHi biofilm development and stability. We have utilized *in vitro* static and continuous flow models of biofilm development by NTHi to demonstrate that magnesium cations enhance biofilm formation by NTHi. We found that the divalent cation chelator EDTA is effective at both preventing NTHi biofilm formation and at treating established NTHi biofilms. Furthermore, we found that the matrix destablilizers EDTA and DNaseI increase the susceptibility of NTHi biofilms to ampicillin and ciprofloxacin. Our observations indicate that DNaseI and EDTA enhance the efficacy of antibiotic treatment of NTHi biofilms. These observations may lead to new strategies that will improve the treatment options available to patients with chronic NTHi infections.

## Introduction

Nontypeable *Hemophilus influenzae* (NTHi) is an opportunistic pathogen associated with respiratory and ear infections in both children and adults. It has been especially implicated in acute exacerbations and chronic infections in patients with conditions such as otitis media with effusion, chronic obstructive pulmonary disease (COPD), and cystic fibrosis (Ehrlich et al. 2002; Hall-Stoodley et al. 2006; Starner et al. 2006). Strains of NTHi isolated from patients with cystic fibrosis, otitis media, and COPD have been shown to form biofilms *in vitro* and *in vivo* (Greiner et al. 2004; Starner et al. 2006; Moriyama et al. 2009; Swords 2012).

Biofilms are structured aggregates of bacteria that are encased in a self-produced polymeric matrix (Costerton et al. 1995, 1999). The matrix which holds the bacterial biofilm together is a complex mixture of macromolecules including exopolysaccharides, proteins, and DNA (Sutherland 2001), which are collectively called extracellular polymeric substances (EPS). The EPS matrix is highly hydrated and has various roles, including adhesion of the biofilm to surfaces, cell–cell cohesion, sequestering of toxic substances from the environment, and protection from predators and the host immune system (Costerton et al. 1981; Donlan and Costerton 2002).

Biofilm infections are particularly problematic, as they are not dealt with effectively by the host's immune system and are recalcitrant to treatment with antimicrobials. Indeed, studies have shown that NTHi biofilm cells exhibit increased resistance to killing by antibiotics compared to the resistance exhibited by planktonic cells (Slinger et al. 2006; Starner et al. 2006; Izano et al. 2009). Although the basis of biofilm-associated antibiotic resistance is not fully understood, it is likely that multiple mechanisms operate simultaneously in biofilms to contribute to antibiotic resistance. One such protective mechanism is thought to be the sequestration and restricted penetration of antibiotics through the biofilm matrix (Brown et al. 1988; de Beer et al. 1997; Donlan and Costerton 2002; Fux et al. 2005).

Many studies have demonstrated the roles of calcium, magnesium, and iron divalent cations in promoting cell growth and cell-to-cell adhesion in biofilms. Divalent cations appear to stabilize the biofilm matrix of a variety of organisms by enhancing structural integrity through electrostatic interactions that serve to cross-link the matrix. Divalent cations have also been shown to enhance the interaction of a mucoid strain of *Pseudomonas aeruginosa* with tracheal epithelium (Marcus et al. 1989). In some bacteria, divalent cations also enhance EPS production. For example, mutations in the magnesium up-take system of *Aeromonas hydrophilia* cause a reduction of biofilm formation and swarming motility (Merino et al. 2001) and divalent cations, in particular Mg^2+^, were found to greatly enhance EPS production by *Staphylococcus epidermidis* strains (Ozerdem Akpolat et al. 2003). In contrast, in the presence of the cation chelator ethylenediaminetetra-acetic acid (EDTA), EPS production by *S. epidermidis* was significantly reduced (Ozerdem Akpolat et al. 2003). As divalent cations also contribute to the integrity and stability of the outer membrane of Gram-negative bacteria (Leive and Kollin 1967; Geesey et al. 2000), EDTA likely exerts antimicrobial activity by chelating cations necessary for growth and membrane stability and may also display anti-biofilm activity by reducing EPS production and/or enhancing the detachment of bacterial cells from the biofilm (Banin et al. 2006). EDTA has been shown to have bactericidal activity against both *Staphylococcus aureus* and *P. aeruginosa* biofilm cells and causes dispersal of *P. aeruginosa* cells from the biofilm (Banin et al. 2006). Furthermore, a combination of gentamicin and EDTA has been shown to result in eradication of *P. aeruginosa* biofilms (Banin et al. 2006).

Extracellular DNA (eDNA) has been shown to be required for biofilm development by *P. aeruginosa* (Steinberger et al. 2002; Whitchurch et al. 2002; Matsukawa and Greenberg 2004; Allesen-Holm et al. 2006). Addition of the eDNA degrading enzyme DNase I inhibits biofilm development by *P. aeruginosa* and established *P. aeruginosa* biofilms can be effectively dispersed by treatment with eDNA degrading enzymes (Whitchurch et al. 2002; Nemoto et al. 2003). Interestingly, in *P.aeruginosa*, eDNA has been shown to act as a cation chelator due to its highly anionic character and electrostatic interactions between negatively charged DNA, and cations such as Mg^2+^ and Ca^2+^, are thought to stabilize the eDNA matrix (Mulcahy et al. 2008; Lewenza 2013). Thus it is conceivable that the ability of EDTA to disperse *P. aeruginosa* biofilms occurs, at least, in part through cation sequestration leading to destabilization of the eDNA matrix.

It is now becoming increasingly apparent that eDNA is a major matrix component in biofilms formed by both Gram-positive and Gram-negative bacteria and that the eDNA in the biofilm matrix plays an important role in disease pathogenesis and resistance to antimicrobials (Costerton et al. 1981; Mulcahy et al. 2008; Chiang et al. 2013; Jakubovics et al. 2013; Lewenza 2013). The matrix of NTHi biofilms is comprised largely of eDNA, which constitutes the major volumetric component of NTHi biofilms (Jurcisek and Bakaletz 2007; Izano et al. 2009). Despite their potentially important role in stabilizing the eDNA matrix of NTHi biofilms through electrostatic interactions, divalent cations have not been well studied with regard to their roles in NTHi biofilms. The purpose of this study was to firstly investigate the role of divalent cations in biofilm formation in a clinical isolate of NTHi, when supplied at physiologically relevant concentrations. Secondly, we wanted to identify factors that may contribute to the breakdown of NTHi biofilms by destablilizing the eDNA matrix and to explore the effects of combining these matrix destablilizers with antibiotics to treat NTHi biofilms.

## Methods

### Bacterial strains and culture conditions

The NTHi strain used in this study (strain 502) was obtained from an ear infection and was provided by the Royal Children's Hospital, Melbourne. NTHi 502 was cultured overnight in brain heart infusion (BHI) broth or in chemically defined medium pH 9 (CDM) (Coleman et al. 2003), supplemented with 2 *μ*g/mL nicotinamide adenine dinucleotide (NAD) and 10 *μ*g/mL hemin (sBHI) at 37°C, 5% CO_2_.

### Static biofilm formation assay

Static biofilm formation by NTHi 502 was assessed using a modified microtitre plate assay with crystal violet staining (O'Toole et al. 2000). Briefly, an NTHi 502 overnight culture was sub-cultured 1:100 and grown to an OD_595_ of 0.4 and then diluted 1:100 in CDM. For each condition, triplicates of 100 *μ*L/well were dispensed into wells of a 96-well flat-bottomed tissue culture-treated microtitre plate (Falcon, Becton Dickinson, Franklin Lakes, NJ, USA). The plates were sealed with sterile breathable film (Aeraseal; Excel Scientific, Victorville, CA, USA) and incubated at 37°C, 5% CO_2_. After 24 h incubation, the plates were washed and biofilm biomass stained with 0.2% (w/v) crystal violet, washed again and the crystal violet solubilized with 20% acetone in ethanol. A_595_ of the solubilized crystal violet was assayed as an indication of biofilm biomass.

### Treatment of established static biofilms

For experiments requiring treatment of an established biofilm, static biofilms were cultured as above in 96-well microtitre plates. The wells were washed with sterile H_2_O and CDM containing the various treatments added to each well. Plates were covered with Aeraseal and cultured for one hour at 37°C, 5% CO_2_. After incubation, the plates were washed, stained with crystal violet and biomass quantified as above.

### Microscopy of static biofilms

For microscopy of static biofilms in 96-well plates, biofilms were formed in *μ*Clear® black 96 well polystyrene cell culture microplates (Greiner bio-one, Frickenhausen, Germany) under the required conditions at 37°C, 5% CO_2_. Biofilms were stained with the nucleic acid stain, SYTO9® (Life Technologies Corp, Carlsbad, CA, USA) and visualized microscopically. Confocal laser scanning microscopy images (CLSM) were obtained with a Nikon A1 confocal microscope with Ζ-series images taken in 0.5 *μ*m slices. Biofilms were volume rendered using IMARIS® Software (Bitplane AG, Zurich, Switzerland).

### Flow cell biofilms

NTHi 502 biofilms were formed in continuous flow cells as described previously (James et al. 1995). To inoculate the chambers, an overnight broth culture of NTHi 502 was diluted in CDM to an optical density at 600 nm of 0.1. Inoculation was performed by back flowing the culture in the flow cells assembled with plastic coverslips (ibidi®, Munich, Germany). Media flow was stopped and cultures were left statically for 2 hours at 37°C, 5% CO_2_ to allow bacterial attachment. CDM was diluted to 1% in phosphate-buffered saline (PBS) and flowed through the channels at a rate of 80 *μ*L/min. The flow cell was placed in an incubator at 37°C, 5% CO_2_ and the biofilms formed for 48 hours. Biofilms were fluorescently stained with SYTO9® and visualized microscopically. When applicable, bacterial viability was determined with a live/dead staining solution. Two stock solutions (SYTO9®: 1 *μ*L in 2 mL, propidium iodide: 1 *μ*L in 1 mL) were diluted in PBS and injected into the flow channels. Biofilms were left for 1 h and then washed with PBS and fixed with 4% paraformaldehyde for 1 h. After washing with PBS to remove the fixative, CLSM images were taken with a Nikon A1 confocal microscope with Ζ-series images taken in 0.5 *μ*m slices. For quantitative analysis of biofilms, image stacks were acquired from equivalent areas of the flow channel where the flow of medium was calculated to be laminar. Biofilm images were volume rendered using IMARIS® software.

### Treatment of established flow cell biofilms

In experiments where EDTA, DNaseI or antibiotic treatment was required, 25 mmol/L EDTA, 100 Kunitz units DNaseI (D5025; Sigma Aldrich Co, St Louis, MO, USA), 50 *μ*mol/L ampicillin or 1.2 *μ*mol/L ciprofloxacin were added to 1% CDM in PBS. Biofilms were then treated with EDTA, DNaseI or antibiotics for 1 h. After staining with SYTO9®, CLSM images were taken with a Nikon A1 confocal microscope with Ζ-series images taken in 0.5 *μ*m slices. Biofilms were volume rendered using IMARIS® software.

### Antimicrobial susceptibility testing

Minimal inhibitory concentrations (MICs) were determined using a microtitre dilution method. Briefly, overnight cultures of bacteria were diluted to a final concentration of 10^5^ cells/mL. Samples were added to equivalent volumes of various concentration of antibiotics, EDTA and DNaseI distributed on microtitre plates. After 24 h of incubation at 37°C, 5% CO_2_, the MIC was recorded as the lowest concentration of drug in which there was no visible growth.

Similarly, minimal biofilm eradication concentration (MBEC) and minimal biofilm inhibitory concentration (MBIC) were calculated for biofilms grown on microtitre plates for 24 h. MBIC was defined as the minimal concentration of treatment required to completely inhibit biofilm development. MBEC was defined as the minimal concentration of treatment required to eradicate the biofilm after 1 h of treatment.

### Checkerboard assay for assessing fractional inhibitory concentration index

To evaluate the interaction of EDTA and the two antibiotics used in this study, ampicillin and ciprofloxacin, the fractional inhibitory concentration index (FICI) was calculated for each combination. A checkerboard assay was set up in microtitre plates with the following conditions. Doubling dilutions of a single antibiotic (amp = 100–6.25 *μ*mol/L) (ciprofloxacin = 10–1.25 *μ*mol/L) were dispensed into successive rows and stepwise dilutions of EDTA (100–3.125 mmol/L) in successive columns (Lewis et al. 2002). The fractional inhibition concentration index (FICI) was calculated for each combination using the following formula:


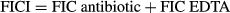


where the FIC of the antibiotics is the MBEC of each antibiotic in combination/MBEC of antibiotic alone and FIC of EDTA is the MBEC of EDTA in combination/MBEC of EDTA alone. Similarly, FIC values were calculated for planktonic cells. MIC values were used for planktonic cells instead of MBEC values. The results were interpreted as follows: ≤0.5, synergistic; >0.5 to ≤4, additive; and >4, antagonistic (Odds 2003).

### Statistics

Data are presented as mean ± SEM. Mean values were compared by an unpaired two-tailed Student's *t*-test for two groups and *P* < 0.05 was considered significant. Quantification of biofilms was performed using COMSTAT software (Heydorn et al. 2000).

## Results

### Divalent cations have differential affects on NTHi biofilm formation

**Figure 1:**
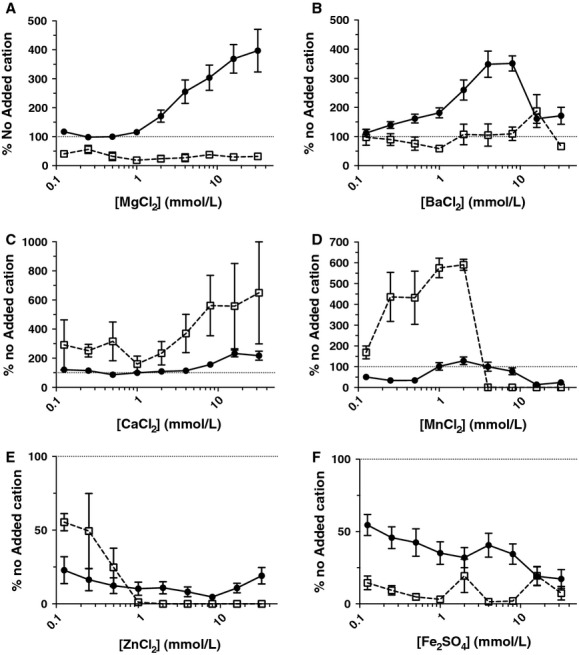
NTHi 502 static biofilm biomass and planktonic cell viability in the presence of divalent cations. Biofilms were formed in the presence of twofold increases in concentration of Mg^+^ (A), Ba^2+^(B), Ca^2+^(C), Mn^2+^(D), Zn^2+^(E), and Fe^2+^(F) cations in CDM for 24 h at 37°C, 5% CO_2_. Circles represent biofilm biomass. Squares represent planktonic cell growth. Planktonic cell viability was measured by CFU counts and biofilm biomass quantitated with A595 measurement of crystal violet staining. Graphs show values relative to no added cation control. Dotted line indicates 100% of no added cation control values. In the presence of Fe^2+^, Mn^2+^, and Zn^2+^, biofilm formation and planktonic cell viability generally decreased as the cation concentration increased. Biofilms were increased in the presence of Ba^2+^ and Ca^2+^ in conjunction with an increase in planktonic cell viability. Mg^2+^ was the only cation to result in increasing biofilm formation without an increase in planktonic cell viability. NTHi, nontypeable *Hemophilus influenzae*; CDM, chemically defined medium pH 9.

In this study, six divalent cations (Fe^2+^, Mn^2+^, Ca^2+^ Zn^2+^, Ba^2+^, and Mg^2+^) were investigated for their effects on static biofilm formation by a clinical NTHI isolate by measuring biofilm biomass in 96-well microtitre tray assays. To determine the influence of divalent cations on the viability of planktonic cells, colony-forming units were also determined. These assays showed that addition of Fe^2+^, Mn^2+^, and Zn^2+^ inhibited biofilm formation and decreased planktonic cell viability (Fig.1). This suggests that these cations were likely to be toxic to the planktonic cells and that this accounted for the observed inhibition of biofilm development. In contrast, the addition of Ba^2+^ and Mg^2+^ resulted in enhanced biofilm formation, whereas Ca^2+^ only caused a mild enhancement of biofilm formation at high concentration (Fig.1). The increase in biofilm biomass in the presence of Ca^2+^ and Ba^2+^ was associated with an increase in planktonic cell growth, suggesting that enhanced growth may account for the observed increase in biofilm biomass. However, the presence of Mg^2+^ resulted in a considerable increase in biofilm biomass, and was associated with a decrease in planktonic cell numbers relative to the control media that had no added Mg^2+^. This suggests that Mg^2+^ may be inducing NTHi biofilm formation and in doing so reduces the numbers of planktonic cells remaining in the growth medium.

**Figure 2:**
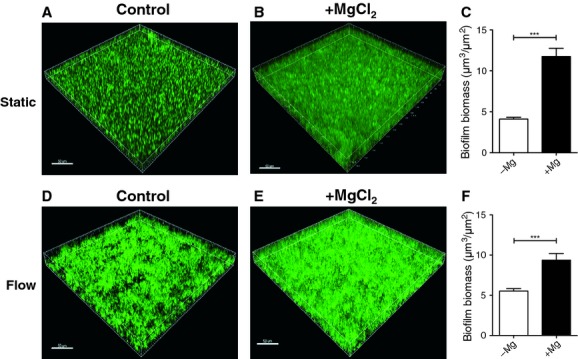
Mg^2+^ enhances NTHi biofilm formation. Biofilms were formed by NTHi 502 in CDM in the absence (A and D) or presence (B and E) of 2.5 mmol/L Mg^2+^ using static (A–C) or flow cell (D–F) models of NTHi biofilm develpment. Biofilms were stained with SYTO9® and imaged by CLSM (A, B, D, E). Biofilm biomass was quantitated from CLSM images with COMSTAT (C and F). Scale bar 50 *μ*m. NTHi, nontypeable *Hemophilus influenzae*; CDM, chemically defined medium pH 9; CLSM, confocal laser scanning microscopy.

To further explore the role of Mg^2+^ in enhancing biofilm development, biofilms formed under static conditions were examined using CLSM. In these assays the effect of adding 2.5 mmol/L Mg^2+^ was assessed as this is the concentration of Mg^2+^ found in human serum (Swaminathan 2003). Biofilms were stained with SYTO9® and visualized using CLSM (Fig2). Under static conditions, in the absence of added Mg^2+^, biofilm microcolonies were sparsely distributed across the coverslip surface (Fig.2A). However, with the addition of 2.5 mmol/L Mg^2+^, tower-like structures were present (Fig.2B) and the biomass was enhanced by ∼threefold as compared to the control biofilms formed in the absence of added Mg^2+^ (Fig.2C).

We also examined the effect of Mg^2+^ on NTHi biofilm formation under flow conditions. In the presence of 2.5 mmol/L Mg^2+^, the NTHi biofilms were thicker and denser than those formed without added Mg^2+^ (Fig.2D and E). Quantitative analysis of the flow biofilms showed an ∼twofold increase in biofilm biomass when Mg^2+^ was added to the media (Fig.2F).

### Cation chelation reduces NTHi biofilm biomass

Our observations have shown that addition of Mg^2+^ enhances NTHi biofilm formation. We hypothesized that by removing these divalent cations through chelation, the formation of NTHi biofilms would be inhibited and that established biofilms would be dispersed. A concentration range of 0.097–50 mmol/L of the divalent cation chelator EDTA was tested in the static biofilm assay using a nutrient medium containing 2.5 mmol/L Mg^2+^. Under these assay conditions, the minimum inhibitory concentration (MIC) of EDTA for planktonic growth was determined to be 7.5 mmol/L, whereas the minimum biofilm inhibitory concentration (MBIC) of EDTA was 12.5 mmol/L.

**Figure 3:**
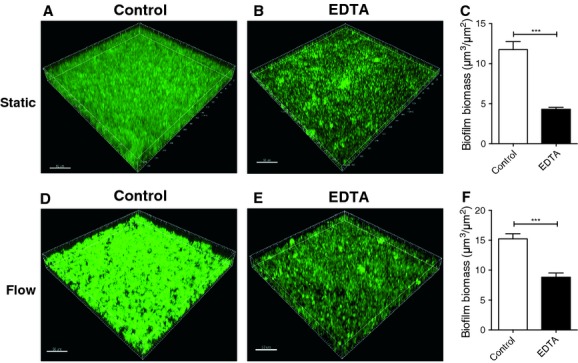
Cation chelators treat established biofilms. To determine the ability of chelators to treat established biofilms, biofilms were formed using static (A–C) or flow (D–F) models of biofilm development, in media supplemented with 2.5 mmol/L Mg^2+^. Biofilms were then treated for 1 h with 25 mmol/L EDTA (B and E) or received no treatment (A and D). Biofilms were stained with SYTO9® and imaged by CLSM (A, B, D, E). Biofilm biomass was quantitated from CLSM images with COMSTAT (C and F). Scale bar 50 *μ*m. CLSM, confocal laser scanning microscopy, EDTA, ethylenediaminetetra-acetic acid.

The efficacy of EDTA in treating established biofilms was examined using CLSM. Biofilms were formed in a static mode for 24 h and EDTA at twice the MBIC concentration (25 mmol/L) was used to treat biofilms for 1 h. Biofilms were stained with SYTO9® and imaged using CLSM. An established static biofilm that was not treated with EDTA displayed a layer of attached cells with scattered tower-like biofilm structures (Fig.3A). In contrast, biofilms that were treated with 25 mmol/L EDTA had no tower-like structures and a reduced attached cell layer (Fig.3B). Quantitative analysis of the biofilm biomass revealed a reduction of ∼threefold when static biofilms cultured for 24 h were treated with 25 mmol/L EDTA for 1 h (Fig.3C).

We also used a flow biofilm model to examine the effect that EDTA has on mature NTHi biofilms cultured for 48 h. Treatment with 25 mmol/L EDTA for 1 h resulted in a thinner and less structured biofilm (Fig.3E), with a 42% biomass reduction (Fig.3F) as compared to untreated NTHi biofilms.

### EDTA enhances antibiotic efficacy against NTHi biofilms

Biofilms are notoriously resistant to a wide range of antibiotics, including *β*-lactams and fluoroquinolones (Walters et al. 2003). Indeed, NTHi biofilms have been previously shown to have an increased resistance to *β*-lactam antibiotics (Slinger et al. 2006). We hypothesized that inclusion of EDTA with antibiotics may increase the efficacy of antibiotics against NTHi biofilms by increasing permeability of the matrix and/or reducing sequestration, thereby enabling better penetration of the antibiotic into the biofilm. Our observations suggest that EDTA may be an effective NTHi biofilm matrix destabilizer. We therefore tested this in combination with two antibiotics, ampicillin and ciprofloxacin, to determine if EDTA increased the efficacy of these antibiotics against established NTHi biofilms.

To investigate whether EDTA and ampicillin or ciprofloxacin have synergistic, additive or antagonistic behaviors, fractional inhibition concentration index (FICI) values were calculated for EDTA in combination with ampicillin and ciprofloxacin, utilizing the respective MBEC (the minimum biofilm eradication concentration) antibiotic values or MIC (minimum inhibitory concentration for planktonic growth) antibiotic values as reference points.

The MBEC values were found to be 2.5 *μ*mol/L for ciprofloxacin, 12.5 *μ*mol/L for ampicillin, and 30 mmol/L for EDTA. We found that the combination of EDTA with ciprofloxacin against biofilms resulted in an FICI value of 2.71 while EDTA in combination with ampicillin resulted in an FICI value of 1.41 against biofilms. As these FICI values were between 0.5 and 4, the interaction of EDTA with ciprofloxacin and ampicillin are considered to be additive for biofilm cultures.

The MIC values against planktonic cells were found to be 1.25 *μ*mol/L for ciprofloxacin, 6.125 *μ*mol/L for ampicillin and 12.5 mmol/L for EDTA. We found that EDTA in combination with ciprofloxacin resulted in an FICI value of 2.81 while EDTA in combination with ampicillin resulted in an FICI value of 3.9 against planktonic cells.

These results show that MBEC values were twice that of the MIC values for ciprofloxacin, ampicillin, and EDTA in isolation. The combination of EDTA with ciprofloxacin or ampicillin was additive against both biofilms and planktonic cultures of NTHi 502 although the high FICI value for EDTA with ampicillin (3.9) against planktonic cells is close to the borderline for antagonistic interactions (FICI >4).

**Figure 4:**
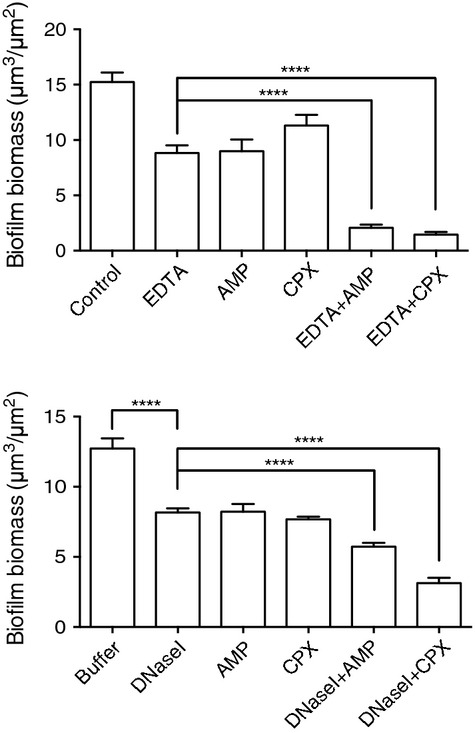
EDTA or DNaseI enhance the efficacy of ampicillin or ciprofloxacin treatment of established biofilms. Biofilms were formed in the flow cell model of NTHi biofilm development and treated with 25 mmol/L EDTA alone or in combination with either 50 *μ*mol/L of ampicillin or 1.2 *μ*mol/L ciprofloxacin (upper) or with DNaseI (100 Kunitz U/mL) alone or in combination with either 50 *μ*mol/L of ampicillin or 1.2 *μ*mol/L ciprofloxacin (lower). Biofilm biomass was quantitated from CLSM images with COMSTAT. Data presented as mean ± SEM (****P < 0.0001). NTHi, nontypeable *Hemophilus influenzae*; CLSM, confocal laser scanning microscopy, EDTA, ethylenediaminetetra-acetic acid.

Confocal laser scanning microscopy (CLSM) was then conducted on biofilms established for 48 h under flow conditions and treated for 1 h with either 50 *μ*mol/L ampicillin alone, 25 mmol/L EDTA alone, or EDTA and ampicillin combined (Fig.4). Quantitative analysis revealed that EDTA reduced biofilm biomass by 31% and ampicillin treatment reduced biofilm biomass by 41%. The combination of EDTA and ampicillin was more effective, reducing biofilm biomass by 86% compared to the untreated control (Fig.4A). Similar experiments using 1.2 *μ*mol/L ciprofloxacin showed that ciprofloxacin alone reduced the biofilm biomass by 26% while the combination of EDTA and ciprofloxacin resulted in the most effective treatment with a reduction of 90% of biofilm biomass when compared to the control (Fig.4A).

**Figure 5:**
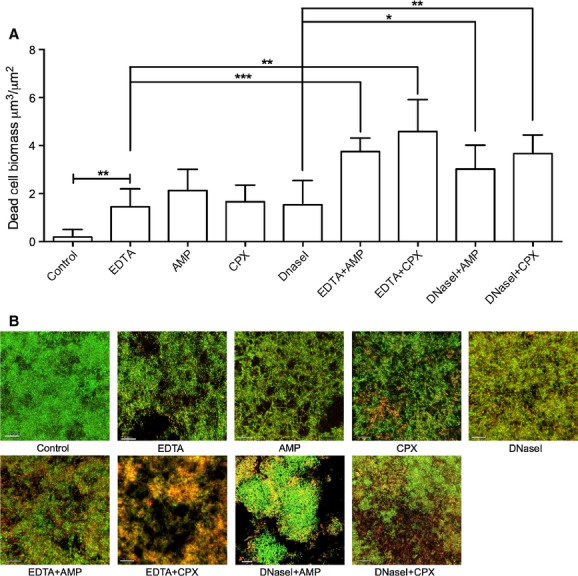
Effect of EDTA and DNaseI in combination with antibiotics on biofilm cell viability. Biofilms were formed in the flow cell model of NTHi biofilm development and treated with EDTA or DNaseI, alone or in combination with ampicillin or ciprofloxacin. Bacterial viability was assessed with live/dead staining kit and imaged using CLSM. Quantitative analysis of dead cell biomass was performed using COMSTAT of the proridium idodide channel (A). Data presented as mean ± SEM (***P < 0.001, **P < 0.01, *P < 0.05). Representative images of live/dead stained biofilms are shown (B). Scale bar 100 *μ*m. NTHi, nontypeable *Hemophilus influenzae*; CLSM, confocal laser scanning microscopy, EDTA, ethylenediaminetetra-acetic acid.

Live/dead staining of NTHi 502 biofilms revealed that the highest biomass of dead cells was achieved when EDTA was combined with ciprofloxacin with a 25-fold increase in dead cells biomass when compared to the control biofilm (Fig.5).

### Effect of DNaseI on NTHi biofilms

As eDNA has been previously shown to be the major matrix component in NTHi biofilms (Jurcisek and Bakaletz 2007), a possible explanation for our observations is that the Mg^2+^ cations serve to strengthen the eDNA matrix through electrostatic interactions with the negatively charged eDNA as has been shown previously with *P. aeruginosa* biofilms (Mulcahy et al. 2008; Lewenza 2013).

We have determined that when NTHi 502 biofilms are cultured for 48 h in flow conditions and then treated with DNaseI, that this results in a 30% reduction in biofilm biomass. These observations suggest that eDNA is a major component of the matrix of biofilms formed by NTHi strain 502. Therefore, it is possible that inclusion of Mg^2+^ to the biofilm culture media enhances biofilm development, at least in part, by strengthening the eDNA matrix through electrostatic interactions and that cation chelation with EDTA serves to weaken the eDNA matrix.

We therefore performed combinatorial experiments with DNaseI and antibiotics to determine if DNaseI had a similar effect as EDTA at enhancing antibiotic efficacy against NTHi 502 biofilms and planktonic cells. We found that the MBEC for DNaseI was 100 Kunitz U/mL. Surprisingly, the MIC for DNaseI against planktonic cultures of NTHI 502 was also 100 Kunitz U/mL which suggests that DNaseI alone affects NTHi 502 viability.

We found that the combination of DNaseI with ciprofloxacin against biofilms resulted in an FICI value of 1.05 while DNaseI in combination with ampicillin resulted in an FICI value of 1.25 against biofilms. As these FICI values were between 0.5 and 4, the interaction of DNaseI with ciprofloxacin and ampicillin are considered to be additive for biofilm cultures. We found that DNaseI in combination with ciprofloxacin resulted in an FICI value of 2.13 whereas DNaseI in combination with ampicillin resulted in an FICI value of 4.58 against planktonic cells. As was observed with EDTA, the combination of DNaseI with ciprofloxacin was additive against both biofilms and planktonic cultures of NTHi 502. However, the combination of DNaseI with ampicillin was additive against biofilms but antagonistic against planktonic cells.

CLSM was then conducted on biofilms established for 48 hours under flow conditions and treated for 1 h with antibiotics and DNaseI alone or in combination (Fig.4B). These experiments showed that DNaseI had a very similar effect as EDTA at enhancing antibiotic efficacy against NTHI biofilms. Ampicillin combined with DNaseI decreased biofilm biomass by 54% and ciprofloxacin combined with DNaseI, resulted in biofilm biomass reduction of 75% (Fig.4B). Live/dead assays revealed that DNaseI treatment alone resulted in dead cell biomass similar to treatments with EDTA or antibiotics alone suggesting that DNaseI treatment reduces NTHi 502 biofilm cell viability.

## Discussion

Previous studies have shown that NTHi biofilms exhibit increased resistance to killing by antibiotics compared to planktonic cells (Slinger et al. 2006; Starner et al. 2006; Izano et al. 2009). In this study we have investigated the role of divalent cations in NTHi biofilm formation. To gain an understanding of how divalent cations affect NTHi biofilm formation, we tested the effect of six different divalent cations on NTHi biofilm formation and planktonic cell growth. Most of the divalent cations tested resulted in dramatically decreased biofilm biomass as well as decreased planktonic cell numbers at the majority of concentrations. This strongly suggests that these cations were toxic to the planktonic cells, and therefore biofilms were unable to be formed. The remaining three cations tested, Ba^2+^, Ca^2+^, and Mg^2+^ resulted in an overall increase in biofilm biomass.

Of particular interest was the increase in biofilm biomass seen in the presence of Mg^2+^, as Mg^2+^ was the only divalent cation tested that increased biofilm biomass dramatically without increasing planktonic cell growth. This suggested that the increases in biomass in the presence of Mg^2+^ were due to a direct affect on biofilm formation. Importantly, we found that inclusion of Mg^2+^ at physiological concentrations significantly increased NTHi biofilm biomass. While the influence of Mg^2+^ on biofilm formation by NTHi has not been previously investigated, researchers have found that Mg^2+^ enhances biofilm formation in a number of other bacterial species including *A. hydrophilia* and *Pseudomonas fluorescens* (Merino et al. 2001; Song and Leff 2006). Furthermore, in a study conducted in mixed biofilms of *P. aeruginosa* and *Klebsiella pneumoniae*, the authors concluded that electrostatic interactions created in the presence of divalent cations contribute to biofilm cohesion (Chen and Stewart 2002).

Divalent cations are known to be important for bacterial cell division and cell wall integrity (Gray and Wilkinson 1965; Asbell and Eagon 1966). As they have also been shown to play a role in biofilm formation, it is not surprising that cation chelators have been tested for the prevention and treatment of biofilm formation in many bacterial species. In particular, the use of the divalent cation chelator EDTA in preventing biofilm formation, especially in catheter-related infections has become an exciting area of research. Indeed, EDTA has been approved as a treatment of biofilms in humans as a component of catheter lock solution (Hancock and Wong 1984). EDTA has been shown to have an effect on at least 11 bacterial biofilm-forming species, including those formed by *P. aeruginosa*, *S. aureus*, and MRSA (Raad et al. 2002). EDTA in conjunction with the antibiotic monocycline has been shown to be highly effective against *S. epidermidis*, *S. aureus*, and *Candida albicans* biofilm on the surface of catheters (Raad et al. 2002). Prolonged treatments with EDTA can be lethal to free-living proteobacteria (Gray and Wilkinson 1965; Leive 1974; Hancock 1984; Banin et al. 2006), and short treatments can increase the permeability of the outer membrane to hydrophobic molecules (Leive 1974; Nikaido and Vaara 1985). Here, we have shown for the first time that EDTA has the ability to prevent biofilm formation and remove established biofilms of a clinical NTHi isolate. CLSM of biofilms formed under flow conditions revealed that EDTA treatment of established biofilms resulted in decreased biofilm biomass.

As eDNA has been shown to be a major component of the extracellular matrix in NTHi biofilms (Jurcisek and Bakaletz 2007), it is likely that the anionic nature of eDNA binds positively charged magnesium cations, thereby strengthening the biofilm matrix. We hypothesized that matrix destabilizers in combination with antibiotics would be more effective than the single agents alone as the matrix destabilizers may enhance accessibility of these antibiotics into the cell clusters (Stewart 2003). Indeed, in *P. aeruginosa* biofilms eDNA has been shown to shield the cells against aminoglycosides by delaying their penetration (Chiang et al. 2013).

In this study, we found that established NTHi 502 biofilms were extremely vulnerable to ampicillin and ciprofloxacin when combined with EDTA or DNaseI. We have not determined the mechanism via which these effects occur, but one possibility is that these may be due to destabilization of the eDNA matrix, thereby enabling greater penetration of the antibiotic into the biofilm either through increased penetration into cell clusters or via reduced sequestration into the matrix.

Interestingly, we observed that both EDTA and DNaseI reduced the viability of planktonic and biofilm cells, which suggests that at least some of the observed effects could be due to direct action on the bacterial cells by these agents. The finding that DNaseI is toxic to NTHi 502 was surprising as we and others have shown previously that DNaseI is not bactericidal against *P. aeruginosa* (Whitchurch et al. 2002) or *Burkholderia cepacia* complex (Messiaen et al. 2014).

Further understanding of the mechanisms associated with NTHi biofilm cohesion and antibiotic resistance could lead to the development of biofilm-specific agents for the management and treatment of NTHi biofilm-associated infections. Our observations suggest that DNaseI and EDTA enhance the efficacy of antibiotic treatment of NTHi biofilms. As both DNaseI and EDTA are currently approved for human use, these observations may lead to new strategies that will improve the treatment options available to patients with chronic NTHi infections. However, the recent report that, unlike the effect observed in *P. aeruginosa*, DNaseI treatment does not increase susceptibility of *B. cepacia* complex species to tobramycin (Messiaen et al. 2014) indicates that the efficacy of the combination therapies such as those described in our study are likely to be highly dependent on the antibiotic and species involved.

## References

[b1] Allesen-Holm M, Barken KB, Yang L, Klausen M, Webb JS, Kjelleberg S (2006). A characterization of DNA release in *Pseudomonas aeruginosa* cultures and biofilms. Mol. Microbiol.

[b2] Asbell MA, Eagon RG (1966). Role of multivalent cations in the organization, structure, and assembly of the cell wall of *Pseudomonas aeruginosa*. J. Bacteriol.

[b3] Banin E, Brady KM, Greenberg EP (2006). Chelator-induced dispersal and killing of *Pseudomonas aeruginosa* cells in a biofilm. Appl. Environ. Microbiol.

[b4] de Beer D, Stoodley P, Lewandowski Z (1997). Measurement of local diffusion coefficients in biofilms by microinjection and confocal microscopy. Biotechnol. Bioeng.

[b5] Brown MR, Allison DG, Gilbert P (1988). Resistance of bacterial biofilms to antibiotics: a growth-rate related effect?. J. Antimicrob. Chemother.

[b6] Chen X, Stewart PS (2002). Role of electrostatic interactions in cohesion of bacterial biofilms. Appl. Microbiol. Biotechnol.

[b7] Chiang WC, Nilsson M, Jensen PO, Hoiby N, Nielsen TE, Givskov M (2013). Extracellular DNA shields against aminoglycosides in *Pseudomonas aeruginosa* biofilms. Antimicrob. Agents Chemother.

[b8] Coleman HN, Daines DA, Jarisch J, Smith AL (2003). Chemically defined media for growth of *Haemophilus influenzae* strains. J. Clin. Microbiol.

[b9] Costerton JW, Irvin RT, Cheng KJ (1981). The bacterial glycocalyx in nature and disease. Annu. Rev. Microbiol.

[b10] Costerton JW, Lewandowski Z, Caldwell DE, Korber DR, Lappin-Scott HM (1995). Microbial biofilms. Annu. Rev. Microbiol.

[b11] Costerton JW, Stewart PS, Greenberg EP (1999). Bacterial biofilms: a common cause of persistent infections. Science.

[b12] Donlan RM, Costerton JW (2002). Biofilms: survival mechanisms of clinically relevant microorganisms. Clin. Microbiol. Rev.

[b13] Ehrlich GD, Veeh R, Wang X, Costerton JW, Hayes JD, Hu FZ (2002). Mucosal biofilm formation on middle-ear mucosa in the chinchilla model of otitis media. JAMA.

[b14] Fux CA, Costerton JW, Stewart PS, Stoodley P (2005). Survival strategies of infectious biofilms. Trends Microbiol.

[b15] Geesey GG, Wigglesworth-Cooksey B, Cooksey KE (2000). Influence of calcium and other cations on surface adhesion of bacteria and diatoms: a review. Biofouling.

[b16] Gray GW, Wilkinson SG (1965). The effect of ethylenediaminetetra-acetic acid on the cell walls of some gram-negative bacteria. J. Gen. Microbiol.

[b17] Greiner LL, Watanabe H, Phillips NJ, Shao J, Morgan A, Zaleski A (2004). Nontypeable *Haemophilus influenzae* strain 2019 produces a biofilm containing N-acetylneuraminic acid that may mimic sialylated O-linked glycans. Infect. Immun.

[b18] Hall-Stoodley L, Hu FZ, Gieseke A, Nistico L, Nguyen D, Hayes J (2006). Direct detection of bacterial biofilms on the middle-ear mucosa of children with chronic otitis media. JAMA.

[b19] Hancock RE (1984). Alterations in outer membrane permeability. Annu. Rev. Microbiol.

[b20] Hancock RE, Wong PG (1984). Compounds which increase the permeability of the *Pseudomonas aeruginosa* outer membrane. Antimicrob. Agents Chemother.

[b21] Heydorn A, Nielsen AT, Hentzer M, Sternberg C, Givskov M, Ersboll BK (2000). Quantification of biofilm structures by the novel computer program COMSTAT. Microbiology.

[b22] Izano EA, Shah SM, Kaplan JB (2009). Intercellular adhesion and biocide resistance in nontypeable *Haemophilus influenzae* biofilms. Microb. Pathog.

[b23] Jakubovics NS, Shields RC, Rajarajan N, Burgess JG (2013). Life after death: the critical role of extracellular DNA in microbial biofilms. Lett. Appl. Microbiol.

[b24] James GA, Korber DR, Caldwell DE, Costerton JW (1995). Digital image analysis of growth and starvation responses of a surface-colonizing Acinetobacter sp. J. Bacteriol.

[b25] Jurcisek JA, Bakaletz LO (2007). Biofilms formed by nontypeable *Haemophilus influenzae* in vivo contain both double-stranded DNA and type IV pilin protein. J. Bacteriol.

[b26] Leive L (1974). The barrier function of the gram-negative envelope. Ann. N. Y. Acad. Sci.

[b27] Leive L, Kollin V (1967). Controlling EDTA treatment to produce permeable *Escherichia coli* with normal metabolic processes. Biochem. Biophys. Res. Commun.

[b28] Lewenza S (2013). Extracellular DNA-induced antimicrobial peptide resistance mechanisms in *Pseudomonas aeruginosa*. Front. Microbiol.

[b29] Lewis RE, Diekema DJ, Messer SA, Pfaller MA, Klepser ME (2002). Comparison of Etest, chequerboard dilution and time-kill studies for the detection of synergy or antagonism between antifungal agents tested against Candida species. J. Antimicrob. Chemother.

[b30] Marcus H, Austria A, Baker NR (1989). Adherence of *Pseudomonas aeruginosa* to tracheal epithelium. Infect. Immun.

[b31] Matsukawa M, Greenberg EP (2004). Putative exopolysaccharide synthesis genes influence *Pseudomonas aeruginosa* biofilm development. J. Bacteriol.

[b32] Merino S, Gavin R, Altarriba M, Izquierdo L, Maguire ME, Tomas JM (2001). The MgtE Mg2 +  transport protein is involved in *Aeromonas hydrophila* adherence. FEMS Microbiol. Lett.

[b33] Messiaen AS, Nelis H, Coenye T (2014). Investigating the role of matrix components in protection of *Burkholderia cepacia* complex biofilms against tobramycin. J. Cyst. Fibros.

[b34] Moriyama S, Hotomi M, Shimada J, Billal DS, Fujihara K, Yamanaka N (2009). Formation of biofilm by *Haemophilus influenzae* isolated from pediatric intractable otitis media. Auris Nasus Larynx.

[b35] Mulcahy H, Charron-Mazenod L, Lewenza S (2008). Extracellular DNA chelates cations and induces antibiotic resistance in *Pseudomonas aeruginosa* biofilms. PLoS Pathog.

[b36] Nemoto K, Hirota K, Murakami K, Taniguti K, Murata H, Viducic D (2003). Effect of Varidase (streptodornase) on biofilm formed by *Pseudomonas aeruginosa*. Chemotherapy.

[b37] Nikaido H, Vaara M (1985). Molecular basis of bacterial outer membrane permeability. Microbiol. Rev.

[b38] Odds FC (2003). Synergy, antagonism, and what the chequerboard puts between them. J. Antimicrob. Chemother.

[b39] O'Toole G, Kaplan HB, Kolter R (2000). Biofilm formation as microbial development. Annu. Rev. Microbiol.

[b40] Ozerdem Akpolat N, Elci S, Atmaca S, Akbayin H, Gul K (2003). The effects of magnesium, calcium and EDTA on slime production by *Staphylococcus epidermidis* strains. Folia Microbiol.

[b41] Raad I, Hachem R, Tcholakian RK, Sherertz R (2002). Efficacy of minocycline and EDTA lock solution in preventing catheter-related bacteremia, septic phlebitis, and endocarditis in rabbits. Antimicrob. Agents Chemother.

[b42] Slinger R, Chan F, Ferris W, Yeung SW, St Denis M, Gaboury I (2006). Multiple combination antibiotic susceptibility testing of nontypeable *Haemophilus influenzae* biofilms. Diagn. Microbiol. Infect. Dis.

[b43] Song B, Leff LG (2006). Influence of magnesium ions on biofilm formation by *Pseudomonas fluorescens*. Microbiol. Res.

[b44] Starner TD, Zhang N, Kim G, Apicella MA, McCray PB (2006). *Haemophilus influenzae* forms biofilms on airway epithelia: implications in cystic fibrosis. Am. J. Respir. Crit. Care Med.

[b45] Steinberger RE, Allen AR, Hansa HG, Holden PA (2002). Elongation correlates with nutrient deprivation in *Pseudomonas aeruginosa*-unsaturates biofilms. Microb. Ecol.

[b46] Stewart PS (2003). Diffusion in biofilms. J. Bacteriol.

[b47] Sutherland IW (2001). The biofilm matrix – an immobilized but dynamic microbial environment. Trends Microbiol.

[b48] Swaminathan R (2003). Magnesium metabolism and its disorders. Clin. Biochem. Rev.

[b49] Swords WE (2012). Nontypeable *Haemophilus influenzae* biofilms: role in chronic airway infections. Front. Cell. Infect. Microbiol.

[b50] Walters MC, Roe F, Bugnicourt A, Franklin MJ, Stewart PS (2003). Contributions of antibiotic penetration, oxygen limitation, and low metabolic activity to tolerance of *Pseudomonas aeruginosa* biofilms to ciprofloxacin and tobramycin. Antimicrob. Agents Chemother.

[b51] Whitchurch CB, Tolker-Nielsen T, Ragas PC, Mattick JS (2002). Extracellular DNA required for bacterial biofilm formation. Science.

